# The CareVirtue Digital Journal for Family and Friend Caregivers of People Living With Alzheimer Disease and Related Dementias: Exploratory Topic Modeling and User Engagement Study

**DOI:** 10.2196/67992

**Published:** 2024-12-24

**Authors:** Andrew C Pickett, Danny Valdez, Lillian A White, Priya Loganathar, Anna Linden, Justin J Boutilier, Clover Caldwell, Christian Elliott, Matthew Zuraw, Nicole E Werner

**Affiliations:** 1 Department of Health & Wellness Design School of Public Health- Bloomington Indiana University Bloomington, IN United States; 2 Department of Applied Health Science School of Public Health- Bloomington Indiana University Bloomington, IN United States; 3 University of Wisconsin-Madison Madison, WI United States; 4 University of Ottawa Ottawa, ON Canada; 5 Whiplash Technology, Inc San Diego, CA United States

**Keywords:** caregiving, dementia, social support, technology, intervention, Alzheimer disease, family, care network, elder, CareVirtue, open text, online platform, digital journaling tool, computational informatics, thematic analysis, topic modeling, neurodegeneration, gerontology, sentiment analysis

## Abstract

**Background:**

As Alzheimer disease (AD) and AD-related dementias (ADRD) progress, individuals increasingly require assistance from unpaid, informal caregivers to support them in activities of daily living. These caregivers may experience high levels of financial, mental, and physical strain associated with providing care. CareVirtue is a web-based tool created to connect and support multiple individuals across a care network to coordinate care activities and share important information, thereby reducing care burden.

**Objective:**

This study aims to use a computational informatics approach to thematically analyze open text written by AD/ADRD caregivers in the CareVirtue platform. We then explore relationships between identified themes and use patterns.

**Methods:**

We analyzed journal posts (n=1555 posts; 170,212 words) generated by 51 unique users of the CareVirtue platform. Latent themes were identified using a neural network approach to topic modeling. We calculated a sentiment score for each post using the Valence Aware Dictionary and Sentiment Reasoner*.* We then examined relationships between identified topics; semantic sentiment; and use-related data, including post word count and self-reported mood.

**Results:**

We identified 5 primary topics in users’ journal posts, including descriptions of specific events, professional and medical care, routine daily activities, nighttime symptoms, and bathroom/toileting issues. This 5-topic model demonstrated adequate fit to the data, having the highest coherence score (0.41) among those tested. We observed group differences across these topics in both word count and semantic sentiment. Further, posts made in the evening were both longer and more semantically positive than other times of the day.

**Conclusions:**

Users of the CareVirtue platform journaled about a variety of different topics, including generalized experiences and specific behavioral symptomology of AD/ADRD, suggesting a desire to record and share broadly across the care network. Posts were the most positive in the early evening when the tool was used habitually, rather than when writing about acute events or symptomology. We discuss the value of embedding informatics-based tools into digital interventions to facilitate real-time content delivery.

## Introduction

### Background

Nearly 7 million people in the United States currently live with Alzheimer disease (AD) or AD-related dementias (ADRD), a number that is projected to double by 2050 [1]. AD/ADRD generally progresses slowly; however, as disease symptomology worsens, most people living with AD/ADRD require support from informal caregivers (ie, unpaid family or friends) [1-5]. Recent estimates suggest these caregivers provide roughly 16 billion hours of unpaid care labor annually in the United States. Caring for people living with AD/ADRD is highly complex and has broad psychological, physical, and economic consequences [1,4,6]. Care partners are often underresourced and unsupported [5-8], which has been associated with high levels of care partner stress, burden, burnout, depression, morbidity, and isolation [2,3,9]. The national plan to address AD highlights the need to expand support for people with AD/ADRD and their families [10]. This research aimed to explore the use of a novel care support platform, CareVirtue, by AD/ADRD caregivers, particularly focusing on specific aspects of use for the platform’s care journal feature.

### Caregiving for Individuals Living With AD/ADRD

AD/ADRD can present with a broad set of behavioral and medical symptoms across an extended time period, contributing to the challenges of caregiving [1,11,12]. Yet, caregivers often remain underresourced and overburdened due to the complexity of AD/ADRD care [7]. Compared with caregivers of people living with other chronic conditions, AD/ADRD caregivers report higher levels of burden, characterized by greater financial, emotional, and physical strain associated with care, aiding with a larger number of activities of daily living, and more difficulty maintaining their own health and well-being [1,7]. Further, a recent review found that many behavioral symptoms of AD/ADRD (eg, sleep disturbances, aggression, and care recipient depression) were directly associated with higher levels of caregiver burden [2]. Not surprisingly, and important to this study, higher levels of perceived burden are associated with increased unplanned hospitalization and poorer quality of life for caregivers [1,2,13,14].

Alleviating suboptimal outcomes (eg, caregiver burden) associated with the progressively changing needs of people living with AD/ADRD often requires a network of multiple care partners including extended family, friends, respite providers, and paid in-home care [4,6,9,15,16]. These care networks engage with and support care partners and the person living with AD/ADRD [4,6,8,9,16-18]. Previous research has advanced a foundational understanding of AD/ADRD care networks demonstrating that (1) many AD/ADRD care partners distribute caregiving in a heterogeneous care network [17]; (2) network members have varying levels of contributions that influence the person living with AD/ADRD and other care partners [8,9,17]; and (3) care networks are currently under supported, leading to factors associated with increased caregiver burden, including role conflict and ambiguity among care network members, communication and coordination challenges, and increased task demands on care partners [4-6,16-21].

### Technology Interventions to Support Caregivers

#### Overview

Systematically connecting and activating care networks through technology interventions is a critical area of need for improving caregiver outcomes [8,22]. As rates of AD/ADRD, and associated need for care, are expected to significantly increase in coming decades, there are increasing calls for scalable interventions to support caregivers, particularly those enabled by technology. There is growing evidence that internet-based tools are an effective and appropriate tool for delivering interventions for older adults, including individuals with some cognitive impairment [23,24]. Further, novel informatics-based tools offer potential capacity as a design feature of internet-based interventions, allowing for real-time participant monitoring and modification to meet user’s unique needs in the moment. That is, by embedding certain tools into the design of such interventions, processes for delivering content can be automated to occur in real time [25]. However, to date, there is less research related to technology-based network interventions for AD/ADRD caregivers.

#### The CareVirtue Platform

CareVirtue is a web-based platform designed to connect a care network of AD/ADRD caregivers around the care of a person living with AD/ADRD [22]. CareVirtue is designed with features for documenting, communicating, and coordinating care needs. CareVirtue’s central feature is the care journal in which caregivers can create posts about their daily caregiving experiences, create posts about caregiving updates and logistics, and ask questions that are shared with their care network. Results from a recent feasibility test of CareVirtue indicated that AD/ADRD care networks use CareVirtue’s journal feature to document and share information, acquire information, cocreate strategies, and lend support to the care partner [22]. We also found that care networks used the journal to give and receive logistic, social, and emotional support [26]. Additionally, posting in the journal provided individual benefits to caregivers, including the ability to process emotions through posting in the care journal [26].

Given that the initial feasibility results indicate that journaling in CareVirtue has benefits for AD/ADRD caregivers [22], an important next step is to glean design requirements from actual use patterns that can be used to support and sustain engagement with the care journal. Therefore, this study first sought to apply exploratory topic modeling to identify primary themes among journal posts. We then explored relationships between these computer-generated themes and certain post characteristics (ie, word count and text sentiment score). Finally, we explored potential differences in care journal post characteristics based on time of day and caregiver mood, self-reported at the time of posting.

## Methods

### Ethical Considerations

This study is a secondary analysis of the CareVirtue feasibility study (R41AG069607), which was approved by the University of Wisconsin-Madison Institutional Review Board. All participants provided informed consent. Participants were provided compensation via e-gift card for completing each stage of the trial (totaling US $150). All data was deidentified prior to analysis, with personally identifying information removed. To protect participant information, any names or proper nouns represented in text are pseudonyms.

### Study Design and Primary Study Procedures

The primary study enrolled 51 caregivers and their network members to use CareVirtue for 60 days. Details of the primary study and its findings are provided elsewhere [22].

**Figure 1:**
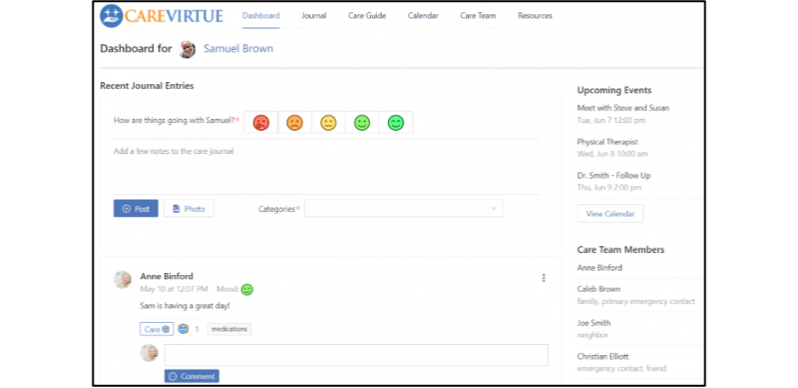
CareVirtue journal.

Care partners who were enrolled in the primary study first created an account on CareVirtue and then invited their chosen care network members to join their CareVirtue account. Care network members included family members and friends who were involved in the care of the person living with AD/ADRD, wanted information about the daily care of the person living with AD/ADRD, or provided social support to the care partner. Care network members can also include paid care partners such as respite care providers and in-home aides and nurses. Features of the CareVirtue platform include a personal care guide, calendar, geolocated resources, and the care journal [22,26]. As noted, this study focused specifically on CareVirtue’s care journal feature. A typical CareVirtue user uses the care journal feature to document and share their daily caregiving experiences and the status of the person living with AD/ADRD with their care network, as well as to communicate with care network members. In addition to posting content using the journal, users are prompted with each post to provide a mood rating using faces as depicted in Figure 1, as well as to assign a relevant “Categories” tag that labels the post for easier search and filter (eg, medication management, behavior related, activities, and hobbies). Journal posts are displayed in the dashboard and shared with all care network members. Anyone in the care network can comment on the journal posts and respond to other network member comments.

### Participants

Participants for this research (n=51) were AD/ADRD caregivers, recruited to participate in a feasibility study for the CareVirtue care platform. Participants were recruited for this study through flyers distributed by community partners in Wisconsin and California and through the Wisconsin Alzheimer Disease Research Center.

### Data Collection

Data were collected in the feasibility study in three parts: the enrollment interview, 60-day use period, and postuse interview. During the enrollment interview, a research team member met with the participant; obtained informed consent; and oriented the participant to CareVirtue, including creating an account, inviting a care network member, and making a journal post. Care network members received an invitation email once an invitation was made in the CareVirtue system that oriented them to the study and electronically obtained informed consent. At the end of the enrollment interview, the participant completed a demographic survey via electronic survey link and then the use period began. To capture actual use, participants were instructed to use CareVirtue as they would like during the use period but were asked to log in at least once per day. At the end of 60 days, a research team member met with the participant to conduct an interview focused on acceptability. Participants were provided compensation via e-gift card for completing each stage of the trial (totaling US $150). For this study, we examine user data generated within the platform (ie, journal entries) across the trial period.

### Analysis

#### Overview

Descriptive statistics for participant demographics were calculated. All journal posts made by enrolled care partners were then exported verbatim to .csv files for analysis. Each journal post represented a separate row in the file, with columns of relevant data including user ID, full post text, time of day, and self-reported mood rating.

#### Bidirectional Encoder Representations From Transformers Topic Modeling Tool

Bidirectional encoder representations from transformers topic modeling tool (BERTopic) uses a neural network approach to topic modeling. Unlike other established unsupervised topic modeling algorithms and tools such as latent Dirichlet allocation, BERTopic does not approximate topics within data using probabilities alone. Indeed, BERTopic’s calculation considers pretrained embeddings from one of many transformer models. These transformer models represent a type of neural network architecture where text entries fed through the program are compared against large-scale language data to better approximate content and meaning. For each entry (ie, journal post), BERTopic then calculates an embedding that converts unstructured language data into a fixed-length and continuous vector. These vectors are then further distilled to generate latent topics for a dataset and other natural language processing (NLP) operations, including semantic meaning, sentiments, and relationships.

#### Principal Components Analysis and Visualization

Embeddings and vectors calculated using BERTopic are complex and often difficult to interpret. As such, we can apply dimensionality reduction techniques to better interpret the data while retaining its constituent parts. This study used a principal components analysis for this task. Principal components analysis is commonly applied for NLP analyses. This analysis allowed us to extract a range of possible clusters, or topics, and visualize them accordingly. Once we reduced the dimensionality of our vectors, we applied hierarchical density-based spatial clustering of applications with noise, to identify and name our latent clusters. We used CountVectorizer to tokenize each topic and class term frequency–inverse document frequency to extract topic words for each cluster. These additional analyses allowed us to generate various visualizations for our data to help us identify the relative similarities and differences across our topics.

#### Valence Aware Dictionary and Sentiment Reasoner

Valence Aware Dictionary and Sentiment Reasoner (VADER) is a rule-based lexicon and web-based dictionary that measures the polarity, or sentiment, of words, phrases, and similar text entries. VADER assumes that most words in the English language can be triaged into either positive, negative, or neutral categories. VADER also assumes that certain words may have higher or lower polarity than others, which implicates a wide range of positivity or negativity, such as the word “horrible” being inherently more negative than the word “bad.” Text fed through the VADER lexicon receives a score based on the presence or absence of negatively and positively charged words. Negative VADER values (–0.99 to –0.01) largely reflect lower sentiment, mood, and affect. Positive VADER values (0.01 to 0.99) largely reflect higher sentiment, mood, and affect.

### Other Measures

#### User Mood

User mood was measured using a 5-point, self-report item completed at the time of posting. Moods were visually represented with colorful emoji-style pictures (Figure 1), ranging from a red angry face to a green smiling face.

#### Time of Day

We extracted the time of day at submission for each post to the CareVirtue Journal, which were recorded as Universal Time Coordinated values. We first converted each time to local time based on user’s location at the time of posting. We then assigned posts to 1 of 4 categories as follows: Morning (6 AM-12 PM), Afternoon (12 PM-5 PM), Evening (5 PM-10 PM), and Overnight (10 PM-6 AM).

### Procedure

We analyzed posts generated by caregivers who used CareVirtue between March and May 2021 using an iterative BERTopic analysis in Python. We first preprocessed our data to remove names, numbers, and dates, which we determined would harm the interpretability of our topic model. Once data were preprocessed, we then iteratively applied the neural network NLP tool (ie, BERTopic) to identify prominent topics within journal entries beginning with 5 topics and concluding with 25 topics, in increments of 5 topics. We calculated coherence scores for each of the iterations as a metric of model fit, selecting the model with the highest overall score for final analysis.

All posts in the dataset were then sorted into groups, based on the topic with which they most closely corresponded. Using computer-generated topics as a grouping variable, we then explored differences in word count and post sentiment (ie, VADER scores). We further explored differences in post word count and VADER sentiment, grouped by caregiver mood and the time of day, respectively. Due to violations of normality assumptions in two outcome variables (word count and VADER sentiment score, Shapiro-Wilk test, *P*<.05), group differences were examined using nonparametric Kruskall-Wallace omnibus tests, with Dunn post hoc procedures to test for individual group differences.

## Results

### Participant Demographics

We first calculated descriptive statistics for participant sociodemographics. Table 1 below provides a detailed summary of this information.

**Table 1:** Caregiver sociodemographics.

Characteristic			Primary caregivers (n=51)
**Gender (women), n (%)**			38 (74.5)
**Age (years), mean (SD)**			60.3 (9.8)
**Race and ethnicity, n (%)**			
	Asian	2 (3.9)	
	Black or African American	1 (2.0)	
	Hispanic or Latinx	2 (3.9)	
	Native American or American Indian	1 (2.0)	
	Not reported	1 (2.0)	
	White	44 (86.2)	
**Marital status, n (%)**			
	Married or domestic partnership	37 (72.5)	
	Divorced	11 (21.6)	
	Single or never married	2 (3.9)	
	Widowed	1 (2.0)	
**Education, n (%)**			
	Postcollege education	19 (37.2)	
	4-year college	17 (33.3)	
	Technical school, vocational training, or community college	10 (19.6)	
	High school diploma or equivalent	5 (9.8)	
**Employment, n (%)**			
	Full-time	21 (41.2)	
	Retired	19 (37.3)	
	Part-time	7 (13.7)	
	Not working	4 (7.8)	
**Income (US $), n (%)**			
	>100,000	18 (35.3)	
	40,000-60,000	8 (15.7)	
	Do not wish to answer	8 (15.7)	
	80,000-100,000	6 (13.7)	
	60,000-80,000	4 (11.8)	
	20,000-40,000	2 (3.9)	
	<20,000	1 (2.0)	
**Location, n (%)**			
	Wisconsin	29 (56.9)	
	California	19 (37.2)	
	Illinois	2 (3.9)	
	Virginia	1 (2.0)	
**Location type, n (%)**			
	Urban	42 (82.3)	
	Rural	9 (17.7)	
**Relationship of caregiver to person living with AD/ADRD^a^, n (%)**			
	Child	28 (54.9)	
	Spouse/partner	20 (39.2)	
	Other relative	3 (5.9)	
**Distance to person living with AD/ADRD, n (%)**			
	In household	34 (66.7)	
	<20 min	12 (23.5)	
	>2 h	3 (5.9)	
	20-60 min	2 (3.9)	

^a^AD/ADRD: Alzheimer disease or Alzheimer disease–related dementias.

### Topic Modeling

**Figure 2:**
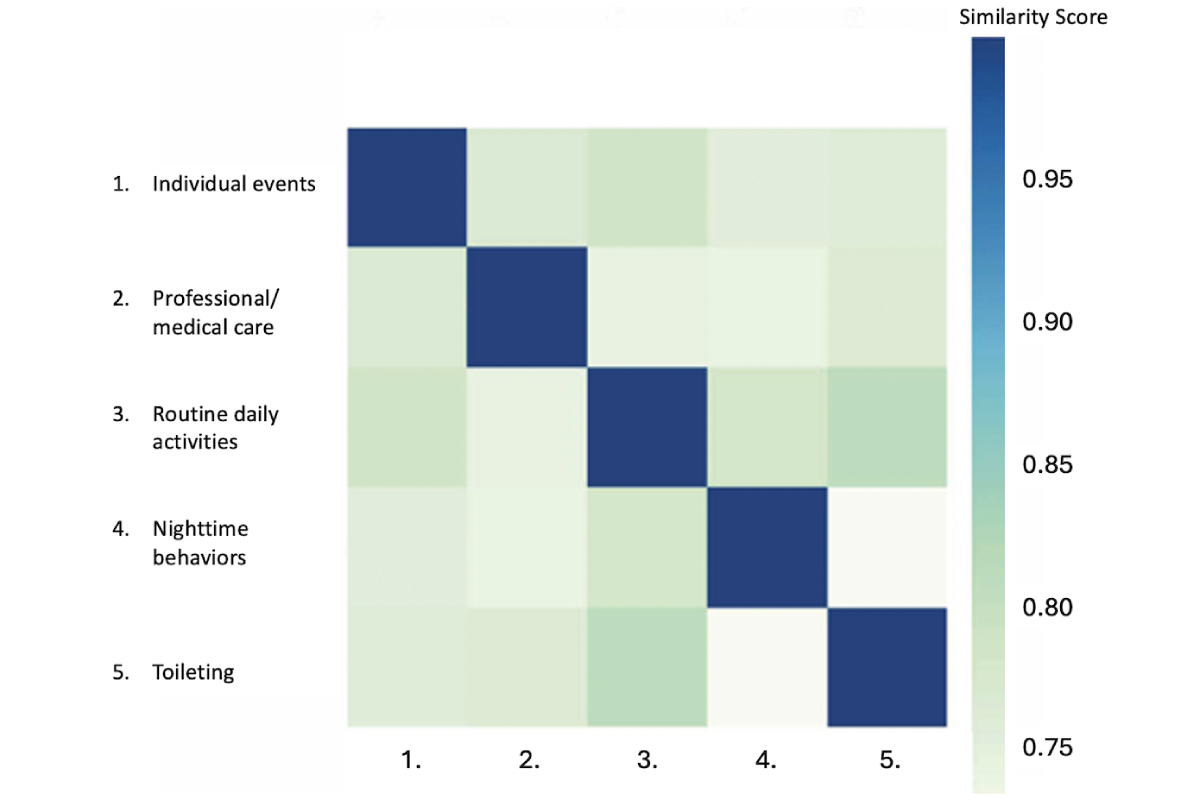
Similarity matrix demonstrating bivariate relationships between topics.

**Figure 3:**
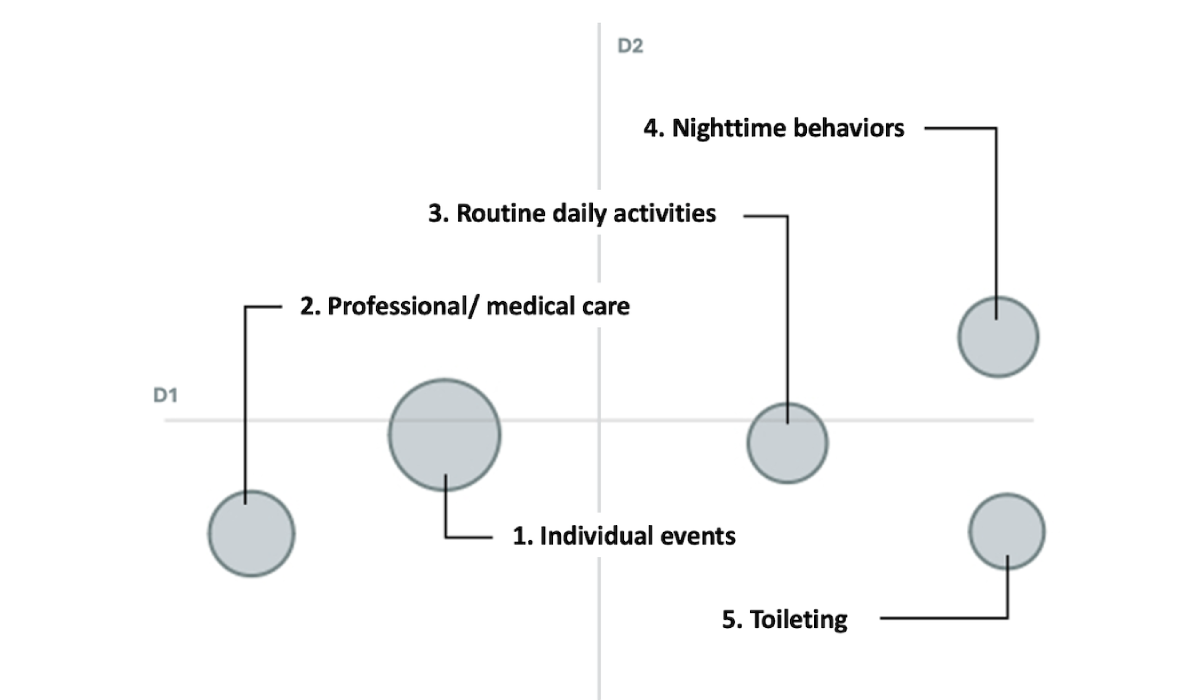
Intertopic distance map visualizing relatedness and relative size of topics observed.

The final dataset comprised the CareVirtue Journal entries (n=1555 posts; 170,212 words) of 51 AD/ADRD caregivers. The optimal model from our analyses suggested 5 latent topics within the corpus. This model was selected after iterative generation of multiple solutions via BERTopic as it had the highest overall coherence score (0.41), indicating adequate fit to the data. After review, we determined the overarching themes to be general content about individual events, professional and medical care, routine daily activities, nighttime symptoms, and bathroom/toileting issues. Table 2 provides further detail about the composition of each topic, including the number of posts (and percentage of the total sample) associated with each topic, mean VADER sentiment score, keywords, and exemplar posts that reflect each topic. Figure 2 is a topic similarity matrix, wherein darker blue colors indicate highly correlated topics; within our data, the 5 latent topics had limited similarity to each other, indicating distinct topics. Figure 3 offers visualization of our findings through an intertopic distance map; intertopic distance maps reflect relative salience and conceptual relationships between topics. Each circle represents a latent topic in the corpus, with larger circles indicating more salient topics. Further, topics are spatially situated such that highly correlated topics will overlap, while conceptually different topics will be arranged further from each other. In our model, no topics overlap, suggesting minimal collinearity between topics. As seen in Figure 2, the topics are loosely clustered, with topics 1 and 2 (individual events and medical care) on one side, and topics 3-5 (routine daily activities, nighttime symptoms, and toileting) clustered together on the other. We discuss each broad cluster in turn below.

On the left of the intertopic distance map, the two largest topics in the corpus were conceptually linked. The largest overall topic (topic 1), with the most associated posts, was related to a broad swath of specific, individual events; this topic was qualitatively the most diverse with respect to overall content. Messages assigned to this topic generally centered on individual (ie, one-time) activities, with particular emphasis on outings or conversations, and included specific details. As such, this topic was largely defined by breaks in standard routine, with many posts focused on conversations or events that were memorable for a novel or specific reason (eg, learned a new tip, spoke to a new person, and tried a new activity). The second most common topic written about by caregivers on the platform is related to various forms of professional and medical care. These included posts about interactions with in-home health aids and nurses, as well as professional care settings (eg, doctors’ offices, nursing homes, assisted living, and memory care). For those discussing in-home professional care, posts were commonly about new instructions for care or updates on patient conditions. Another common subtheme in this topic was the sharing of updates or results from doctor visits, both specific to AD/ADRD (eg, neurologists) and not (eg, dermatologists and dentists).

**Table 2:** Topic model solution for CareVirtue Journal.

Topic ID	Topic name	Post count (n=1555), n (%)	Keywords	Excerpts
1	Individual events	506 (32.75)	Busy, Pastor, Talking, Didn’t, Evening, Banging, Day, Told, Work, Doesn’t	I still try to do things like we did a year ago, but the minute I get there I realize it is hard to get back to the old ways. I keep trying Flea markets, but I can’t keep up with him. He wants to buy everything and talk to everyone, and I always want to leave the minute we get there. I also realized that my friends don’t want to be with him, and I probably have the wrong friends because he is too sweet and good to them. A very interesting day. The one good thing that happened, the small pot turned up. I found it when I went to make popcorn. She would say she never touched it. [She] was hesitant at first, but really got into the painting zoom session with [others]. She was able to visualize the correct colors to use based on the image of the final product. Her control of the paint brushes was good. Lisa plans to offer more sessions in the future.
2	Professional/medical care	296 (19.16)	Aunt, Appointment, Mother, Doctor, Nurse, Care, CAN, Hospice Bed	Was notified by [Name], a caregiver accidentally OVER medicated mom with medication that was NOT hers (Divalproex 125mg in applesauce)! This is the 5th time in 4 months that a medication error has occurred due to caregiver incompetence. Hospice nurse was called and she is being monitored by staff. I called and spoke with [Name] (a CNA in Memory Care) where my Mom resides. She said my Mom is “tooling around” in her wheelchair. She ate all her dinner last night (fish, asparagus, etc), which is great to hear. Her behavior has been good the past couple days. Granny has a new primary care “doctor” — [Name], NP, and she’s great! She is going to do regular home checkups. In her initial visit she completed a basic memory assessment, the first I think that Granny has ever had, and also ordered a visit with a neurologist—something I’ve been trying to make happen for years! I am adding [the NP’s] contact info to the resources tab. Her next visit is scheduled for 5/20.
3	Routine daily activities	258 (16.70)	Nap, Slept Breakfast, Woke, Morning, Lunch Tired, Bed, Sleep Laundry	Friday we did not do showers, so the morning was enjoyable. We went to Walmart to buy dirt for the flower beds. We had lunch, naps and devotions and enjoyed the afternoon as well. [She] ate a good supper, we watched our favorite shows and she went to bed without problem. She slept through the nite with waking up only when I got her up to go to the bathroom. Not much to report. We walked Sunday and Monday and expect to do same daily for the rest of this week. We did Zoom church on Sunday and I played music in the afternoon. Today I played tennis in the morning; [Name] handled laundry. I need to turn on the machine every now and then for her. At dinner she asked four times what was planned for tomorrow, which is very little, pickleball for me. I am waiting for a warmer day, which won’t be this week, apparently, so we can go outside for lunch somewhere, maybe the Cupcake place, which has outdoor picnic tables. The TV experiment isn’t working […]as she can’t figure out the remote. Tuesday, March 30th ~ Today was [dog]’s grooming appointment. Mom was not sure yesterday if she wanted to go with me to have him groomed. She said she was not feeling well and could I go alone. Today, she was ready when I called to say I was coming to pick him up. She had her cash, her keys and the dog ready. We dropped him off and I took her to Walmart for the two items she had on her list. She liked looking at the Easter Candy and decorations. I kept the trip short and then we drove through McDonalds for her chicken sandwich, one of the only foods that I know she will eat, besides sweet pastries and cookies. Everything went well, she likes [pet]’s grooming, and was happy to get out of the house to get groceries. She called at her 7 PM bedtime and said she was checking all the locks again because it was too quiet outside. I assured her it was all okay and she was safe here in this town.
4	Nighttime behaviors	257 (16.63)	Bedtime, Slept, Restless, Sleep, Sleeping, Morning, Agitated, Woke	I had to get up every hour to handle [name]. I need to get steady sleep to function well all day. Up last night for a few hours. She was cold even with electric blanket and extra covers. Last evening until 2 AM this morning, [name] exhibited Sundowners Syndrome symptoms more than ever before. From when she initially went to bed at 5 PM, she was restless, anxious, fearful and attempted to shadow my every movement. Neither one of us slept much until 2 AM. I’m going to give her her bedtime meds later in the hope she will go to bed later in the evening and sleep better from that point forward.
5	Toileting	228 (14.76)	Nurse, Toilet, Doctor, Poop, Bathroom, Walk, Pee, Care, Bed, Change	He has diaper rash back in right groin. put something on it that burned?? arthritis cream? anything in a tube he will use. doesn’t wash well, just took a shower and doesn’t smell good. told him to get in shower and wash off then put on a and d [sic] ointment. will he do?? why is he wearing depends all day? has to go right now! he says. urine the problem? but been in bathroom a lot too for #2 ?? Came home from Grocery store at 8:15 on Sat and the smell of poop reeked throughout the house. [They] tried to clean up the toilet seat but things were smeared. I tried to look in and ask about it but she locked the door! I got in and she was asleep. I didn’t wake her. Well the past 24 hours have been a poop fest. Dad went from being constipated to having loose stools that have been filling his pants. Lots of showers and laundry. I finally had to put a diaper on him this morning. I hope this ends soon but not with constipation again.

The other three topics (ie, topics 3-5) were clustered together to the right of the intertopic distance map. Topic 3 generally outlined daily routines and common activities; spatially, this topic was situated between topic 0 (relating to specific events) and the two final topics related to AD/ADRD behavioral symptomology (ie, topic 4 [nighttime behaviors] and topic 5 [toileting]). Posts associated with topic 3 (routine daily activities) often included a full walkthrough of the caregivers’ day, including any minor events happening throughout the day. This differed from topic 1 in that these posts did not offer details about any single event but tended to be more general in form. Topics 4 and 5 discussed two common challenges associated with AD/ADRD symptomology: nighttime challenges and toileting issues, respectively. In topic 4, caregivers often discussed “sundowning,” which is a state of confusion that often occurs in the late afternoon or early evening and can lead to a disrupted sleep schedule [27]. Users also frequently noted that care recipients would wake often throughout the night, sometimes noting confusion or agitation upon waking. The final topic related to bathroom and toileting issues. Journal entries on this topic often described challenges with incontinence, including secondary infections, dirty laundry, or hesitation around the use of disposable underwear. Some posts also related to challenges with constipation or the care recipient’s refusal to ask for help in toileting.

### Group Differences in Text by Topic, User Mood, and Time of Day

#### Overview

After exploring topics present in user journals, we then sought to better understand group differences in text features (ie, length of post and VADER sentiment score) associated with topic, users’ self-reported mood, and the time of day. Across the whole sample, we found an average of 110.15 (SD 153.41) words per post, with a slightly positive mean VADER sentiment score of 0.23 (SD 0.57).

#### Differences by Topic

Significant group differences across topics were observed with respect to both word count (*χ*^2^_4_=826.11, *P*<.001) and VADER sentiment score count (*χ*^2^_4_=212.37, *P*<.001). Descriptive statistics by topic are presented in Table 3. With respect to word count, all groups were significantly different from each other.

With respect to VADER sentiment, topic 3 about routine daily activities had higher average scores than all other groups. A middle tier, including topics 2 and 5 (professional/medical care and toileting) had the next highest sentiment scores, but did not differ from each other. The lowest tier of scores related to topics 1 and 4 (individual events and nighttime behaviors), which did not significantly differ from each other, but were significantly different from all other groups.

**Table 3:** Descriptive statistics of word count and sentiment score by topic.

Topic	Word count, mean (SD)	VADER^a^ score, mean (SD)
1	33.11 (31.34)	0.11 (0.46)
2	71.97 (60.81)	0.24 (0.52)
3	163.88 (82.34)	0.61 (0.53)
4	46.22 (39.09)	0.07 (0.51)
5	341.93 (258.25)	0.27 (0.57)

^a^VADER: Valence Aware Dictionary and Sentiment Reasoner.

#### Differences by User Mood

Mood ratings were self-reported using nonnumbered emojis at the time of posting (Figure 1). For analysis, mood ratings were numbered from 1 to 5, with 1 representing the worst mood state (angry, red face) and 5 representing the best (happy, green face). We observed differences in both word count (*χ*^2^_4_=46.15, *P*<.001) and sentiment (*χ*^2^_4_=516.93, *P*<.001), when grouped by mood rating. The longest overall posts were found in groups 3 (mean 117.71) and 4 (mean 126.55), which were significantly longer than all other groups, but not significantly different from each other. Word count for groups 1 (mean 91.84), 2 (mean 72.11), and 5 (mean 85.61), did not significantly differ from each other.

**Figure 4:**
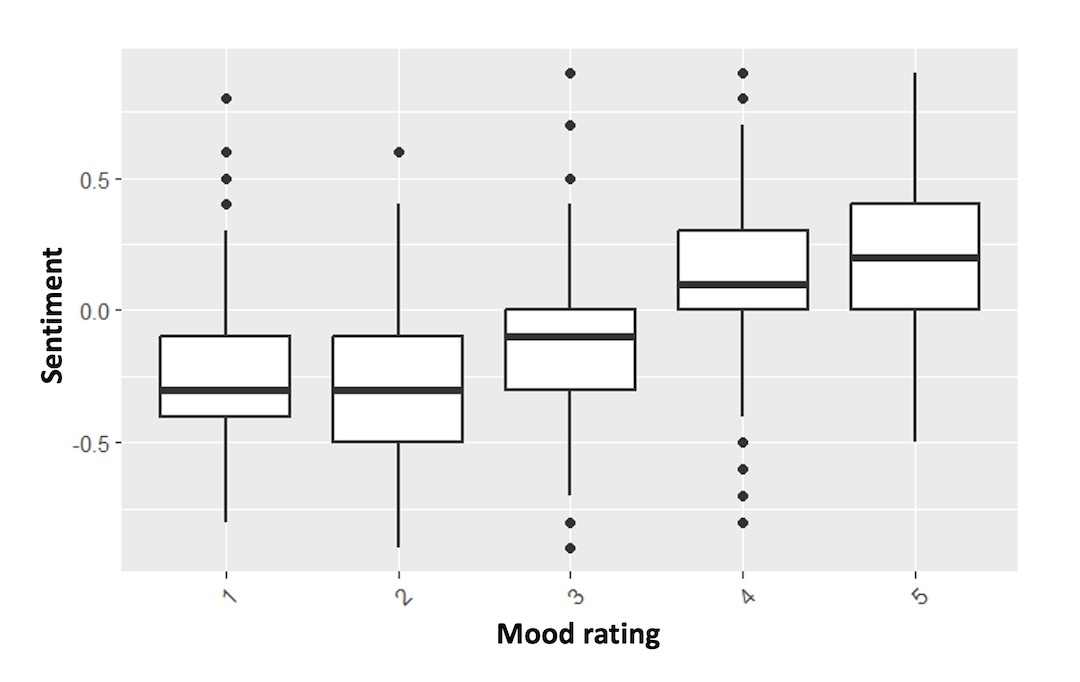
Box-and-whisker plot showing group differences in user sentiment by self-reported mood rating.

Text sentiment (ie, VADER scores) also differed between mood groups, following a roughly linear pattern wherein low user mood posts had the low sentiment scores, while higher user reported mood posts had the highest sentiment scores. This can be seen in Figure 4. The only groups that did not differ significantly from each other were groups 1 and 2.

#### Differences by Time of Day

Finally, we examined differences in word count and sentiment score at different times of day. We again observed significant group differences with respect to both word count (*χ*^2^_3_=56.99, *P*<.001) and sentiment score (*χ*^2^_3_=35.71, *P*<.001). Posts made in the evening (mean 131.05) were statistically significantly longer than all other groups, followed by posts made in the afternoon (mean 110.60) and morning (mean 94.28), which did not significantly differ from each other. Posts made overnight were significantly shorter than all other groups (mean 46.70).

**Figure 5:**
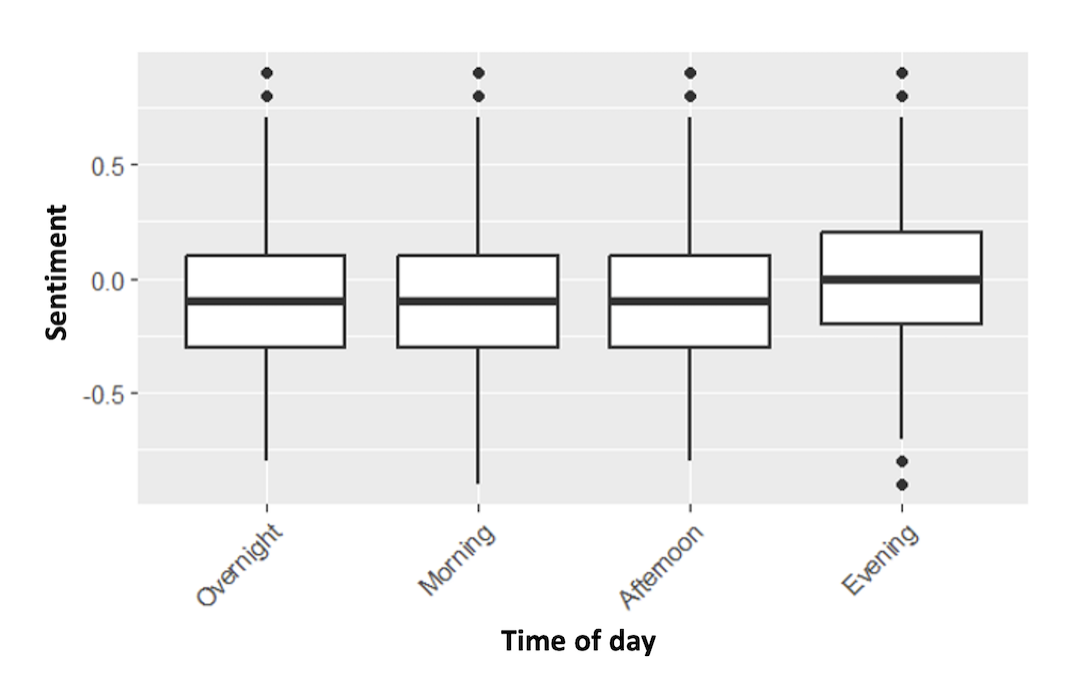
Box-and-whisker plot showing group differences in user sentiment by time of day.

VADER sentiment scores also differed by time of day (Figure 5). Posts made in the evening had significantly higher sentiment than those made at any other time. There were no observed differences in sentiment between other time groups.

## Discussion

### Principal Findings

This study sought to understand the use of the CareVirtue Journal by caregivers of people living with AD/ADRD [22,26], identifying salient topics within the data and exploring patterns related to the topic of discussion, user mood, and time of day. Using an iterative computational approach to identify the optimal topic modeling solution, we identified 5 primary themes in caregivers’ journals. Broadly, these topics related to descriptions of individual events or conversations, professional or medical care, daily activity routines, nighttime symptoms, and toileting behaviors. Across the corpus of posts, users portrayed a generally positive tone, as indicated by a mean VADER score above zero. However, there was a wide deviation in sentiment scores, suggesting there was a variety of content present, including posts that were both highly positive and negative in nature. However, there was a wide deviation in VADER scores, suggesting a wide variety of sentiments across posts in the corpus, including some that were either highly positive or highly negative in nature. In examining post characteristics by time of day, we found posts written in the evening were the most linguistically positive and were longer than posts in all other groups, suggesting a more routine journaling practice at the end of the day. In contrast, the lower sentiment scores and word counts found at other times of day may reflect acute incidents users felt needed to be documented in the moment, or an attempt to fit journal writing into smaller windows of available free time. Broadly, our findings help identify key needs and salient thoughts of AD/ADRD caregivers. These findings have important implications related to increasing support for and improving the well-being of caregivers.

Based on emergent topics in the data, we identified two primary ways users communicated about care tasks and experiences using the Journal feature. These divergent patterns reflect differing approaches or use cases of the Journal feature. The first was defined by sharing information across the care network (ie, much like a social media feed) [17,26], while the other was more individually focused, operating much like a traditional journal for recording personal thoughts and experiences [28,29]. Posts in topic 1 tended to reflect the more social use case of the Journal, often describing a specific event or conversation that was memorable or had useful implications for care. For example, one user wrote, “we did the exersize [sic] class together and [he] was able to participate some of the time. He couldn't answer the question that they asked him about what he is happy about. I guess he is losing his verbal skills.”

In this case, description of this event, and particularly the loss of verbal skill, would be of value to others across the care network as it may influence future interactions with the care recipient. These posts also often reflected somewhat more traditional journaling purposes, where users wrote down their experiences, thoughts, and emotions—engaging in real-time reflection or planning for changes in care practices within the post itself. Likely related to their specificity around a particular need or AD/ADRD-related event, these posts were the shortest in length and had the lowest average sentiment score of any topic.

A second latent topic in our data (ie, topic 3) included generalized discussion around care activities, with users describing an entire day or more of activities [18,29]. Posts in this topic reflected the second use case described above, with users treating the tool much like a traditional written journal. These posts generally offered limited detail about any specific activity; rather, posts on this topic seemed to serve the purpose of cataloging daily routines. Posts on this topic did not generally have specific relevance to care and would likely be of limited use to others in the care network [17]. Broadly, the primary use of these posts by other users would be in day-over-day comparisons of daily activities or symptoms. For example, one post said, “Had a busy day yesterday getting ready for a weekend visitor. Went shopping, dropped things off at recyclers, made use of great weather for a walk. [NAME] was pretty worn out by the end of the day. She went to bed early right after supper. Could not remember eating breakfast or lunch.”

These recap-style posts did not generally include the reflection elements common in topic 1, but rather provided the list of activities without further comment. Despite being among the lengthiest posts in the corpus, this topic had the highest average sentiment score, reflecting a lack of symptom-specific or other negative language that may drive down scores in other topic areas.

Caregivers also used the journal to discuss medical care and other professional assistance related to AD/ADRD care (topic 2) [26]. Posts on this topic were diverse in topic and tone, reflected by the wide deviation in VADER score across the topic. Here, there were posts relaying updates from doctors specifically related to AD/ADRD (eg, neurologists) and other medical providers (eg, dentists and general practitioners). This underscores the nature of AD/ADRD caregiving, which is not limited to a single domain, but often requires many hours per day for a wide range of support tasks [4,5]. Additionally, within this topic, users sometimes discussed individual instances related to professional care settings, including mishaps (eg, missed diagnoses and errors in medication management) and praise for individual providers.

### Implications for Practice

This work has important implications for organizations seeking to provide support for AD/ADRD caregivers. First, our exploratory findings suggest two distinct use cases for the CareVirtue Journal—one for personal journaling and one for shared communication across the care network—in which users engaged roughly equally. Future refinement of care coordination platforms may provide unique space for each, with clearer instruction related to their different purposes [30]. Given the documented importance of care networks in diffusing caregiver burden and the mental health benefits associated with private journaling [17,26,28], we suggest the maintenance of both functionalities. However, due to time constraints associated with care provision, caregiver support interventions should be tailored to achieve specific tasks in a clear and efficient manner. Relatedly, our findings point to the value of both routinized and as-needed engagement with the Journal. We noted that users often posted longer messages, with a more positive sentiment, in the evening likely as part of a daily routine. By contrast, posts made at odd hours were shorter, more negative, and likely related to an acute instance. Again, however, users seemed to value both capacities of the platform. Future intervention development may consider cues to complete routine posts/engagement, while also providing additional tools to maximize use flexibility (eg, mobile optimization) [31].

In reviewing the content posted to users’ Journals, we found a broad set of general topics. However, some AD/ADRD symptomologies were particularly common among posts; specifically, users often posted about sleep and toileting issues [11,12,32]. There was also a unique topic related to professional medical care, both from physicians and in assisted or long-term care facilities. The frequency with which these topics were discussed may indicate particular stress points for caregivers [4]. This is perhaps, not surprising, as these symptoms less likely to be managed by respite or temporary caregivers, who are not with the care recipient after hours or overnight. Prior research has also found that caregivers often face challenges in determining the appropriate timing to move their care recipient into a long-term care facility [33]. Caregiver interventions and organizations may, therefore, focus efforts on creating novel mechanisms for managing these symptoms and making these determinations. For example, digital care coordination platforms like CareVirtue may nudge local, secondary caregivers in the network to explore options for providing overnight care. Broadly, our findings suggest primary caregivers of people living with AD/ADRD experience myriad, complex emotions and challenges associated with care. Novel technology-based interventions are needed to reduce burden and improve caregiver well-being [26].

One important contribution of this work was to demonstrate the capacity to use large language and machine learning tools in understanding user behavior for digital health tools. A prior study used traditional qualitative content analysis to explore the use of the CareVirtue Journal tool [26]. In that study, primary themes that emerged from the data related to caregivers’ information acquisition, information sharing, strategy development, and information feedback. While similar themes emerged in the current analysis, by using computational tools, we were able to derive additional themes and quantitatively relate these themes to user behavior. This is consistent with prior research suggesting the additive value of such methods in understanding and contextualizing large datasets of unstructured text [34,35].

Beyond uses for retrospective data analysis, the informatics-based tools here could be used within the intervention itself to automate processes for delivering real-time content across the care network [36,37]. For example, embedding a calculation of semantic sentiment score (ie, VADER) at the time of posting, given its significant relationship with user mood, could allow for the identification of caregiver stress in real time. This data could be used to then trigger in-program responses, including curated content for the caregiver related to stress reduction strategies, as well as notifications to others in the care network prompting them to reach out to the principal care provider. This is consistent with other digital health interventions, which have begun to embed artificial intelligence and other informatics-based approaches into digital interventions, including artificial intelligence–enabled chatbots [38,39]. Such applications of these tools are increasingly common, given their relatively low cost to implement and high capacity to make digital tools more immediately responsive to user needs [40].

### Limitations and Future Directions

As with all research, this work had certain limitations that limit generalizability and should be noted for consideration. First, this study analyzed the free text responses of AD/ADRD caregivers, who were not given particular prompts or instructions for their use of the Journal tool. As such, individual users perceived differing uses for the CareVirtue Journal feature. While this lack of clarity provided interesting and novel insights for the design process, future research may seek to examine each use case independently. Additionally, those individuals willing to post to the shared Journal space may have certain characteristics (eg, extroversion), which may limit the generalizability of findings to all caregivers. Further, this study was completed with a relatively small sample of predominantly White, female caregivers and may not be generalizable to all caregiving situations. Readers should exercise caution in drawing broad conclusions from this work. Future research may seek to examine Journal use among larger and more diverse samples.

### Conclusions

This study examined the use of a novel tool, the digital CareVirtue Journal, by caregivers of people living with AD/ADRD. Using a computational, informatics approach, we identified 5 salient themes in users’ free-text journal posts. Two related broadly to the caregiving experience, but with unique use cases: a more traditional journaling approach wherein users chronicled daily routines, and a second approach for recording acute or specific events with relevance for others across the care network. Further, caregivers often journaled about professional medical care and two specific symptomologies of AD/ADRD (ie, sleep disturbance and toileting challenges). We found that users who routinely engage the platform in the evening and who reported a better overall mood also tended toward longer and more semantically positive posts. By contrast, users who engaged in off-hours or who reported poor mood tended to write shorter, semantically negative posts. Taken together, these findings suggest multiple, valuable uses for the CareVirtue Journal tool, which offers AD/ADRD caregivers space to reflect and organize their thoughts, as well as to share important care-related information across their networks.

## Abbreviations

AD: Alzheimer disease
ADRD: Alzheimer disease–related dementias
BERTopic: bidirectional encoder representations from transformers topic modeling tool
NLP: natural language processing
VADER: Valence Aware Dictionary and Sentiment Reasoner
